# Syphilitic Rupia

**Published:** 1861-07

**Authors:** 


III. *Syphilitic Rupia.*—F. B., æt 22, remarkably strong, weight 160, was admitted June 11.

In the fall of '59 he contracted a chancre on the inferior and external surface of the prepuce, which was followed in a few days by buboes in *both* groins. These appear to have been indolent and non-suppurating, generally following indurated chancres. For these he was treated in Galveston, Texas, for two or three weeks, and pronounced cured. He has been engaged in the lumber trade in that climate most of the time since, and necessarily exposed in the water very frequently.

About six months since there appeared a bulla upon the superciliary ridge, which soon became purulent and formed a crust, immediately followed by others of like character, until the whole upper part of the face was covered by them. They were of a dirty, brownish color, with ulcerated surfaces beneath. Crops soon appeared upon the body, and more especially upon the arms. As they heal they leave corresponding sized cicatrices of a circular form, wrinkled and extremely puckered in the centre, and of a copper color.

The disease was a well marked syphiloderma, and for some time confined to the face. The most remarkable fact is that his general health was not disturbed in a great degree, he only complaining of a lack of muscular energy whenever the eruption appeared. His skin presents an unnatural dirty brownish tinge, marked by these spots of deeper color, indicating the cicatrization of the ulcers before mentioned. He has now remaining but one crust, which does not increase in size, gives no discharge, as is usual, of ichorous matter, and has an accompanying sore throat or laryngitis, but no ulceration. The epitrochlear, post cervical, and inguinal glands are enlarged. The disease has rapidly yielded to treatment. On account of the otherwise good condition of the patient, so unusual in those afflicted with rupia, the Prof. prescribed the internal use of the prot-iodide of mercury, with the happiest results, instead of the iodide of potass, as is usually recommended. The throat was touched with a two-grain solution of nit. of silver, and he has employed sulphur baths twice or three times a week.

